# Lattice mismatch alleviation in p-CdTe/n-Si heterostructure by surface engineering on Si substrate

**DOI:** 10.1016/j.heliyon.2023.e21536

**Published:** 2023-10-31

**Authors:** Mustapha Isah, Camellia Doroody, Kazi Sajedur Rahman, Muhammad Najib Harif, Tiong Sieh Kiong, Ahmad Wafi Mahmood Zuhdi

**Affiliations:** a Institute of Sustainable Energy (ISE), Universiti Tenaga Nasional (@The Energy University), Jalan Ikram-Uniten, 43000, Kajang, Selangor, Malaysia; b College of Engineering, Universiti Tenaga Nasional (@The Energy University), Jalan Ikram-Uniten, 43000, Kajang, Selangor, Malaysia; c Department of Physics, Kaduna State University, PMB, 2339, Tafawa Balewa Way, Kaduna State, Nigeria; d Solar Energy Research Institute, Universiti Kebangsaan Malaysia, 43600, Bangi, Selangor, Malaysia; e Faculty of Applied Sciences, Universiti Teknologi MARA (UiTM), Cawangan Negeri Sembilan, 72000, Kuala Pilah, Negeri Sembilan, Malaysia

**Keywords:** Energy, CdTe, n-Si, CdTe/Si heterostructure, CdCl_2_ treatment, KOH texturization, Lattice mismatch, Thermal mismatch, Photovoltaics

## Abstract

The study used magnetron sputtering to investigate the growth of cadmium telluride (CdTe) thin films on surface treated n-type silicon (n-Si) substrates. The n-Si substrates were textured using potassium hydroxide (KOH) before the sputter deposition of CdTe. This was followed by cadmium chloride treatment to reduce the strain at the interface of CdTe and Si, which is caused by the incompatible lattice and thermal expansion mismatch (CTE). X-ray diffraction (XRD) analysis showed that the lowest FWHM and dislocation densities were obtained for CdCl_2_/CdTe/txt-nSi, which aligns with the scanning electron microscopy (SEM) results. In the SEM images, the interface bonding between the CdTe and Si surfaces was visible in the cross-sections, and the top-view images revealed sputtered CdTe thin films conforming to the patterns of pyramidal textured Si as an engineered surface to capture more light to maximize absorption in the CdTe/Si tandem design. The Energy dispersive X-ray (EDX) results showed that all the CdTe deposited on textured n-Si exhibited more Te atoms than Cd atoms, irrespective of the CdCl_2_ treatment. The presented results suggest that the texturization and CdCl_2_ treatment improved the morphology and grain boundary passivation of the sputtered CdTe. The adhesiveness of CdTe on the n-Si substrate was also significantly enhanced. Our findings further demonstrate that proper surface treatment of the Si substrate can greatly improve the quality of CdTe grown on Si by reducing the strain that occurs during the growth process. This study demonstrates a valuable method for enhancing the integration of CdTe with Si for two-junction tandem solar cell applications.

## 1 Introduction

The success of cadmium telluride (CdTe) thin film solar cells is seen in its significant commercialization in today's solar cell market. This is primarily due to the high absorption coefficient of cadmium telluride (about 10^5^ cm^−1^), which can absorb 99 % of the AM1.5 solar spectrum at a thickness of just around 1–2 μm [1,2]. CdTe thin-film solar cells also have the advantages of low fabrication cost and stability and can therefore endure harsh weather conditions compared to other solar cell materials. It is the leading commercialised thin film solar cell with a laboratory efficiency of 22.1 % and a record module efficiency of 19.5 % [3,4]. Silicon, on the other hand, is the leading commercialised solar cell globally, with a well-established manufacturing process. So far, manufacturers have achieved module efficiencies of 20.4 % and 24.4 % for multi-crystalline and crystalline Si, respectively [5,6]. The optimum laboratory efficiency achieved for single-junction crystalline Si is about 26.6 % [4], and CdTe and Si are the most successfully commercialised single-junction solar cell technologies; however, they are already approaching their practical and commercial efficiency limits given at around 25–27 % [3,7]. This has triggered the interest of researchers in multijunction or tandem solar cells. The predicted theoretical limit of a two-junction tandem solar cell is approximately 44 %, and the practical limit is approximately 35 % with an optimum combination of band gaps for both the top and bottom cells [[8], [9], [10]]. This shows that the single-junction practical limit can further be extended, therefore increasing the possible attainable practical efficiency of solar cells by multijunction solar cells.

Various material permutations based on group III-V materials have been suggested for multijunction solar cell applications, and their practical and commercial viabilities have been widely studied. These materials include gallium arsenide (GaAs), aluminum gallium indium phosphide (AlGaInP), aluminum indium phosphide (AlInP), indium gallium arsenide (InGaAs), gallium phosphide (GaP), and gallium indium phosphide (GaInP) [7,[11], [12], [13], [14], [15], [16], [17], [18]]. Stacking two or more of these materials monolithically in tandem has shown a promising efficiency of over 35 % [[19], [20], [21], [22]]. However, this high efficiency is usually achieved at a high cost. This restricts their application to high-end users, such as space applications and high-concentration solar systems [14]. The most recent material that has attracted the interest of researchers in tandem solar applications is perovskite solar cell absorber material. This is because of the recent rapid progress made in its research as a solar cell material. In addition, it has a tuneable band gap suitable for tandem solar cell applications that is between 1.48 eV and 2.2 eV [23]. It can be deposited at standard temperature and pressure conditions, making it a low-cost alternative solar cell material. It is usually combined as perovskite/perovskite, perovskite/Si, perovskite/CIGS, perovskite/CdTe, etc., in a tandem structure [[24], [25], [26], [27]]. Recently, a record-breaking efficiency of 27.6 % has been obtained from a perovskite/Si combination using an industrial-produced tunnel oxide passivated contact (TopCon) device [28]. Despite this progress, perovskite has a setback in that it easily transforms into many phases at a temperature of around 65 °C, making it highly unstable in real-time working conditions [29].

Another option yet to be fully explored that has a promising potential of producing high-performance and low-cost tandem solar cells with better stability is by leveraging on the well-established manufacturing processes of both CdTe and Si solar cells. Utilising CdTe and Si in tandem configuration offers an additional advantage such that the CdTe band gap can be tuned from 1.45 eV to around 1.81 eV by adding Zinc (Zn), Magnesium (Mg), or Selenium (Se) to CdTe to form CdZnTe, MgCdTe or CdSe_x_Te_x-1_ alloys [[30], [31], [32]]. The ability to modify the band gap is a distinctive characteristic that allows CdTe solar cells to function as the top cell absorber material in a dual-junction tandem solar cell with a Si bottom cell. The Si band gap (∼1.1 eV is already closely matched with the optimum band gap required for a dual-junction tandem bottom cell absorber material [33,34]. However, one major problem stands in the way of fully developing monolithic CdTe/Si tandem solar cells; that is, there is a lack of a quality method for growing defect-free CdTe or its alloys on Si. Usually, this problem arises because of the large lattice and thermal mismatch between CdTe and Si thin films [35]. Some of the techniques used to deposit CdTe are electrodeposition, thermal evaporation, closed hot-wall epitaxy, close-space sublimation, and magnetron sputtering [[36], [37], [38], [39], [40]]. In this study, the magnetron sputtering technique was utilized to deposit CdTe on pristine and chemically etched Si. Sputtering is used because it enables low substrate deposition temperatures, deposition of a uniform thin film, and reasonable thin-film density [41]. This is advantageous when full CdTe and Si cells are integrated into a tandem solar device. Proper surface preparation of the Si substrate and post-deposition treatments are exploited to improve the quality of the CdTe/Si heterostructure.

## 2 Experiment details

A pristine n-type silicon substrate (pris-nSi) and textured n-type silicon substrate (txt-nSi) were prepared by cutting a double-polished surface n-Si (100) wafer into 3 × 3 cm pieces using a laser cutter. The 3 × 3 cm samples were subjected to RCA cleaning processes to remove both organic and inorganic contaminants by first using a solution of deionized (DI) water: NH_4_OH: H_2_O_2_ at 70 °C for 5 min for RCA1. After rinsing the samples in DI water for 3 min, they were subjected to RCA2 cleaning in a solution of DI water, HCL, and H_2_O_2_ at 70 °C for another 5 min. The samples were then rinsed again by running DI water for 3 min before the final cleaning using a solution of HF: DI-Water (1 ml; 50 ml) for 2 min to remove the native oxides. The samples were then divided into two groups: a set of thoroughly cleaned n-type Si (pris-nSi) and another set of samples that were subjected to wet chemical etching using a mixture of potassium hydroxide (KOH), isopropyl alcohol (IPA), and di-water solution for 10 min at 75 °C to create a fine pyramidal textured Si surface (txt-n Si). All the samples were then cleaned with DI water and dried under nitrogen gas (N_2_).

A 99.999 % pure CdTe target was used in a radio frequency (RF) magnetron sputtering system to deposit CdTe on the pris-nSi and txt-nSi samples at a sputtering power of 50 W and a substrate temperature of 300 °C for 1.5 h. An argon flow of 4 sccm was used, which resulted in maintaining a base and working pressures of 1.5 × 10^−5^ torr and 2.0 × 10^−2^ torr respectively. The said deposition parameters resulted in about 1.5 μm thickness of sputtered CdTe film on the n-Si substrates. Cadmium chloride (CdCl_2_) treatment was then applied to CdTe/pris-nSi and CdTe/txt-nSi. The elemental ratios in the deposited CdTe thin films and the surface and cross-sectional morphologies of the CdTe/pris-nSi and CdTe/txt-nSi heterostructures were characterized by energy-dispersive X-ray spectroscopy (EDS) and Field Scanning electron microscopy (FESEM) using a Bruker QUANTAX EDS-Detector (60 mm^2^) integrated into a Zeiss Merlin FESEM machine. The crystal structure, defect density, and strain induced in the deposited thin films were studied by X-ray diffraction (XRD) using a Bruker DS advance diffractometer.

## 3 Results and discussion

**Fig. 1:**
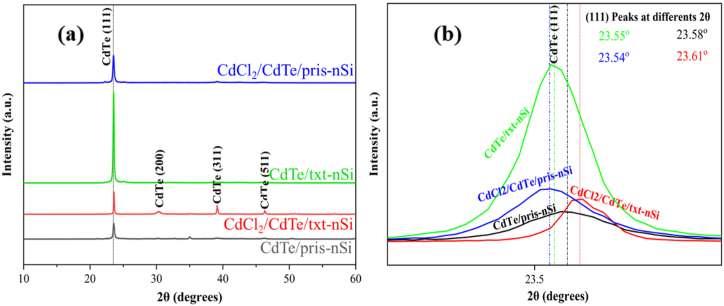
(a) XRD patterns of CdTe crystal planes sputtered on pris-nSi and txt-nSi with their corresponding cadmium chloride (CdCl_2_) treated samples. (b) Shifts in the enlarged image of the (111) peak.

First, XRD analysis was performed to evaluate the changes in the structural characteristics of the four sets of prepared samples. Fig. 1a shows the XRD results of the as-deposited CdTe on a well-prepared and deoxidized pristine n-Si substrate (pris-nSi) and a potassium hydroxide (KOH)-textured n-Si substrate (txt-nSi).

All examined samples of the sputtered CdTe thin films showed strong diffraction peaks at 2θ = 23.58°, 23.54°, 23.55°, and 23.61° for CdTe/pris-nSi, CdCl_2_/CdTe/pris-nSi, CdTe/txt-nSi, and CdCl_2_/CdTe/txt-nSi, respectively, as shown in Table 1. Other peaks of CdTe (200), CdTe (311), and CdTe (511) with lower energies appear for CdCl_2_/CdTe/txt-nSi, as shown in Fig. 1a. All films exhibited a cubic phase of CdTe [JCPDS card No. 01-077-7297]. The strain between the CdTe thin film and the n-Si substrate was analyzed through the shifts in the (111) preferential peak of the sputtered CdTe thin films, as shown by the enlarged peaks in Fig. 1b. The results obtained through calculations and analysis of the XRD data using Equations (1), (2), (3) show a variation in strain and dislocation densities between the four different samples, as shown in Table 1.

**Table 1:** Structural characteristics based on XRD analysis.

Case No.	Sample	Peaks position 2θ (Degree)	FWHM (β)°	D (nm)	Dislocation density δ ( × 10^−4^ nm^−2^)	Microstrain ε( x 10^−3^)
1	CdTe/pris-nSi	23.58	0.2707	31.32	10.190	5.660
2	CdCl_2_/CdTe/pris-nSi	23.54	0.2656	31.91	9.820	5.560
3	CdTe/txt-nSi	23.55	0.1954	43.37	5.321	4.090
4	CdCl_2_/CdTe/txt-nSi	23.61	0.1360	62.32	2.574	2.840

The Scherrer formula given in equation (1) was used to calculate the average crystallite size (D) of the deposited CdTe[42].

(1) $$D=\frac{K\;\lambda }{\beta \;x\;\cos\theta }$$

Where D, is the crystallite size in (nm), K is the Scherrer constant, 0.94 is the K value for spherical crystallite, β is the full width at half maximum (FWHM) in radians (rad), and λ is the wavelength of the x-ray and θ is the Bragg's angle. Meanwhile, the Williamson- Smallman relation given in equation (2) was used to calculate the dislocation density (δ) in nm^−2^[43].

(2) $$\delta =\frac{1}{D^{2}}$$

The microstrain (ε) was estimated with equation (3). All the calculated results are given in Table 1 [44].

(3) $$\varepsilon =\frac{\left(\beta \;x\;\mathrm{Cot}\;\theta \right)}{4}$$

**Fig. 2:**
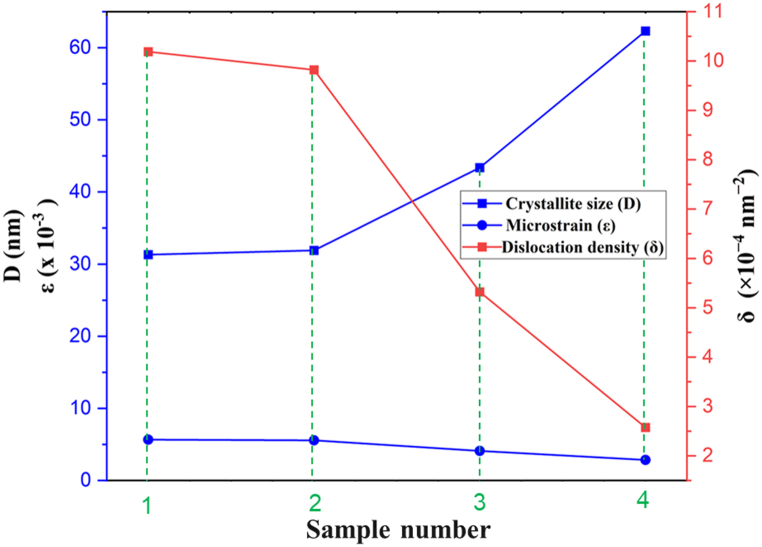
Variation of crystallite size, microstrain and dislocation density for the four different samples deposited.

The CdTe deposited on the pris-nSi substrate (CdTe/pris-nSi) has the most strained interface, which is relaxed by about 2 % of the initial strain after the CdCl_2_ treatment. The CdTe deposited on textured Si and then treated with CdCl_2_ (CdCl_2_/CdTe/txt-nSi) showed about 50 % less compressive stress than the one deposited on untextured and untreated Si substrate CdTe/pris-nSi) while the one deposited on the only textured Si but not treated (CdTe/txt-nSi) has about 28 % less compressive stress than the one deposited directly on the pristine Si substrate. This may be linked to the fact that the textured Si substrate provides more surface area for nucleation to occur, this promotes the thin film growth in a more prepared orientation than in the pris-nSi which has not been textured [45]. Also, CdCl_2_ treatment is known to enhance grain growth and recrystallization of the CdTe thin film through grain boundary passivation [46]. Heat treatment during the CdCl_2_ treatment gives energy for atomic diffusion, making atoms to regroup, thereby forming larger grains [47]. This regrouping of the atoms helps in relaxing the CdTe thin film and hence reduced the lattice mismatch between the sputtered CdTe and the substrate as given in Table 1. The lowest FWHM and dislocation densities were also obtained for CdCl_2_/CdTe/txt-nSi from the XRD data analysis. This indicates that the crystalline quality of the deposited film has improved with the textured and CdCl_2_-treated samples. Fig. 2 shows how both the microstrain and dislocation density decreased with an increased in crystallite size due to the various sample's preparations and treatments.

**Fig. 3:**
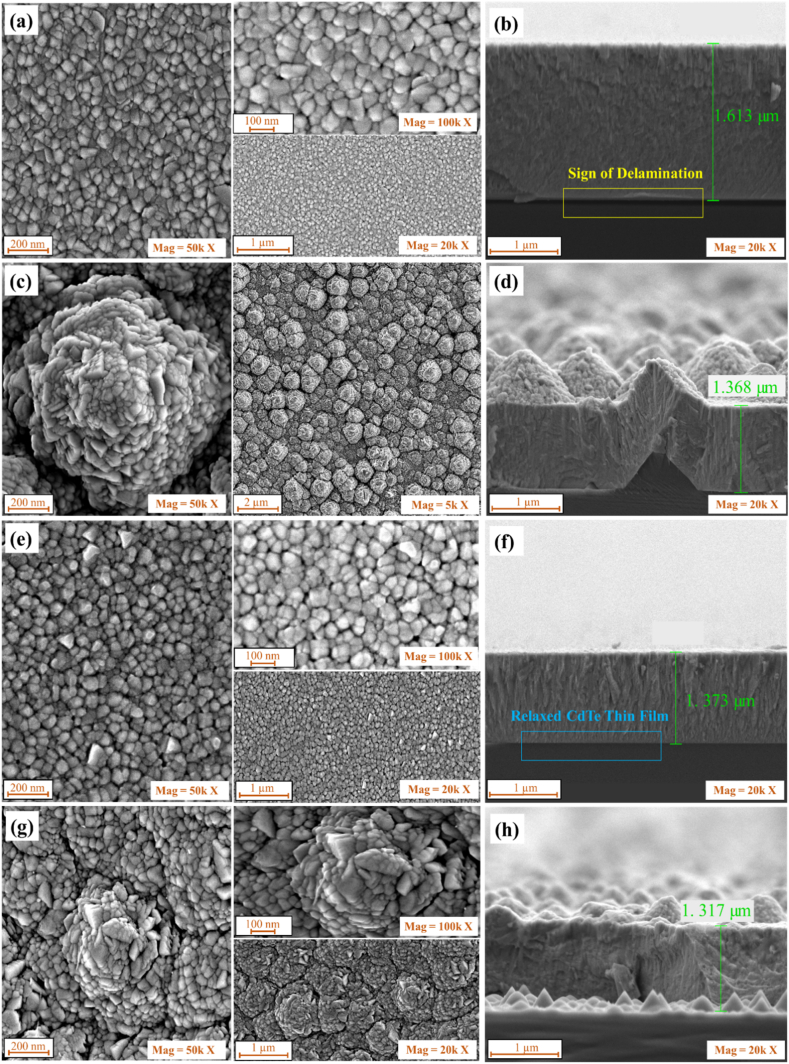
(a) top and (b) cross-section CdTe/pris-nSi, (c) top and (d) cross-section of CdTe/txt-nSi, (e) top and (f) cross-section of CdCl_2_/CdTe/pris-nSi and (g) top and (h) cross-section of CdCl_2_/CdTe/txt-nSi.

SEM images presented in Fig. 3(a) and (b) show the morphological top view and cross-section of CdTe on a pris-nSi with their corresponding CdCl_2_ treated samples. The average grain size obtained is 60 nm. The cross-section shows signs of delamination of the CdTe from the n-Si surface because of the sticking problem usually associated with CdTe and untreated Si surfaces [48] The delamination problem can be seen to be reduced after CdCl_2_ treatment on the CdTe/pris-nSi as shown in Fig. 3f which may be due to the recrystallization and passivation of CdTe grain boundaries as the result of CdCl_2_ treatment process. This also makes the grown CdTe to be more relaxed on the n-Si with average grain size increased to around 70 nm in the CdCl_2_-treated CdTe/pris-nSi as also obtained in the XRD result. Fig. 3 (c) and (d) show the morphology and the cross-section of the deposited CdTe on KOH textured n-Si. This shows good adhesiveness between the CdTe and Si surfaces. The grown CdTe also follows the patterns of the pyramidal textured Si which could be used to trap more light for subsequent absorption by both CdTe and Si when used as tandem heterostructures. Furthermore, as the CdTe/txt-nSi was subjected to CdCl_2_, the grain boundaries become more flattened and realigned, this shows some sign of passivation and recrystallization on the sample as shown in Fig. 3g. However, extra care must be taken to ensure that the roughness due to texturization of the Si substrate does not result in poor quality of the grown thin film. Also, more study of annealing treatment is needed during the CdCl_2_ treatment to assess how it may impact on the Si bottom cell when the two absorber materials are fully integrated as a tandem device.

**Fig. 4:**
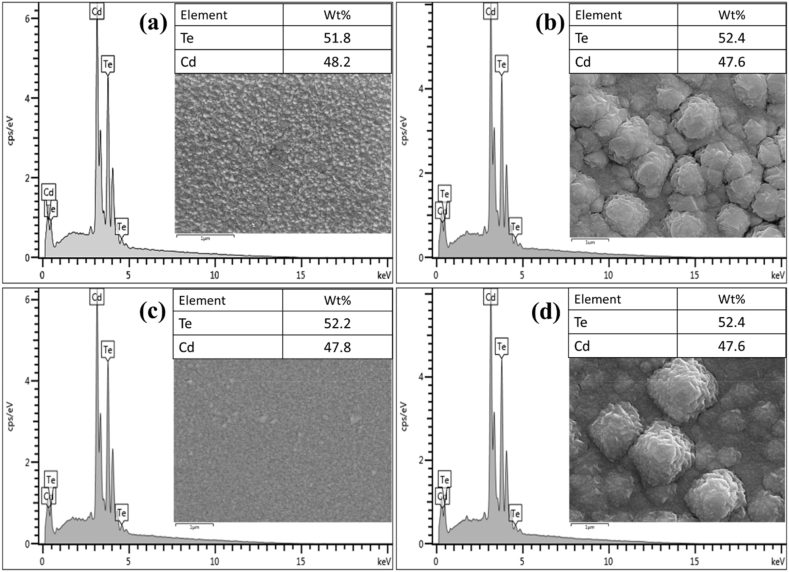
Shows the EDX analysis for CdTe deposited on: (a) pris-nSi (b) CdTe/txt-nSi (c) CdCl_2_/CdTe/pris-nSi and (d) CdCl_2_/CdTe/txt-nSi.

EDX was carried out for all four studied samples to determine the ratios of Cd and Te atoms in them as shown in Fig. 4(a)–(d). The CdTe deposited on the untreated pristine n-Si is richer in Cd atoms than Te atoms while the CdCl_2_ treated CdTe deposited on the pristine n-Si shows an increase in the Te atoms to about 52.2 % from 51.8 %. While all the CdTe deposited on textured n-Si presented a higher Te atom than Cd atoms irrespective of the CdCl_2_.

## 4 Conclusion

CdTe/Si heterostructure was successfully fabricated by proper surface preparations of Si substrate, KOH texturization of Si substrate and subsequent CdTe post-deposition treatment using CdCl_2_. The results show an overall improvement in CdTe crystalline quality from 31.32 nm for the as-grown set of samples to 62.32 nm for the texturized and treated thin films on Si due to the proper surface engineering and post-deposition treatment. The strain induced on the deposited CdTe was shown to be reduced significantly through XRD analysis and SEM images verified the enhancement demonstrating the passivated grain boundaries and relaxed interface between Si and CdTe thin films. Te content in textured samples is presented to be 0.6 to 0.2 % higher for the as grown and treated samples compared to the deposited films on pris-nSi. The reduction of lattice and CTE mismatch as well as defect density in CdCl_2_/CdTe/txt-nSi samples suggest that proper surface preparations, Si texturization by chemical etching, and post-deposition treatment could be promising approaches to fabricate optimal CdTe/Si heterostructure.

## Funding

The authors would like to acknowledge the 10.13039/501100003093Ministry of Higher Education of Malaysia for their support through the HICoE grant no. 2022003HICOE, as well as Dato’ Low Tuck Kwong International Energy Transition Grant under the project code of 202203001ETG. They would also like to express their gratitude to 10.13039/100019523Tenaga Nasional Berhad (TNB) and UNITEN through BOLD Refresh Publication Fund with the project code of J510050002-IC-6 BOLDREFRESH2025 - CENTRE OF EXCELLENCE.

## Data availability

Not applicable.

## Ethical approval

Not applicable.

## CRediT authorship contribution statement

**Mustapha Isah:** Writing – original draft, Visualization, Software, Data curation. **Camellia Doroody:** Writing – review & editing, Visualization, Validation, Methodology, Conceptualization. **Kazi Sajedur Rahman:** Writing – review & editing, Visualization, Validation. **Muhammad Najib Harif:** Writing – review & editing, Validation. **Tiong Sieh Kiong:** Writing – review & editing, Visualization, Validation, Supervision, Resources, Funding acquisition. **Ahmad Wafi Mahmood Zuhdi:** Validation, Resources, Project administration.

## Declaration of competing interest

The authors declare that they have no known competing financial interests or personal relationships that could have appeared to influence the work reported in this paper.
